# Unpacking the mediating effects of language mindset on the relationship between ideal L2 self and learning engagement

**DOI:** 10.3389/fpsyg.2026.1822625

**Published:** 2026-05-20

**Authors:** Xuan Zhou, Youyou Luo, Mingce Huang, T’chiang Ung Chow, Patricia Nora Riget

**Affiliations:** 1 Faculty of Languages and Linguistics, Universiti Malaya, Kuala Lumpur, Malaysia; 2 School of Foreign Studies, Jingchu University of Technology, Jingmen, China

**Keywords:** engagement, language mindset, motivation, second language acquisition, structural equation modeling

## Abstract

**Objectives:**

Recent studies in positive psychology have witnessed an increasing trend examining the influence of learning engagement in second language acquisition (SLA). Numerous antecedent studies reported that learners’ motivation beliefs and language mindsets facilitated learning engagement. However, sporadic studies have attempted to investigate the effects of ideal L2 self on engagement with language mindsets as mediators.

**Methods:**

Adopting a structural equation modelling method, the study modeled the mediating effects of language mindset on ideal L2 self and learning engagement among a total of 431 undergraduates from a university located in central China.

**Results:**

The results are as follows: (1) ideal L2 self and growth mindset positively predict learners’ behavioral, emotional, and cognitive engagement; (2) fixed mindset does not show statistically significant predictive effects on engagement and ideal L2 self; (3) growth mindset mediates the relationship between ideal L2 self and the three subscales of learning engagement.

**Conclusion:**

The study highlights the synergy between motivation and language mindset in boosting engagement and offers pedagogical guidance to both educators and language learners seeking to enhance teaching and learning engagement.

## 1 Introduction

Second language acquisition (SLA) is a lengthy and complex process permeated with considerable challenges, which requires learners to be behaviorally, emotionally, and affectively engaged to achieve desirable learning outcomes (Xu and Feng, 2024). Learning engagement has been considered “the holy grail of learning” (Sinatra et al., 2015). Highly engaged language learners tend to display sustained endeavors and continuous passion for learning goals (Li and Yuan, 2024), as well as stronger motivational orientations toward better future selves (Sun et al., 2024). Additionally, many studies have confirmed the influence of engagement on L2 achievement (e.g., Jiang et al., 2024; Li and Yuan, 2024; Luan et al., 2025). Given the crucial role of engagement in language learning, it becomes necessary to further explore the antecedents that lead to high levels of engagement.

Ideal L2 self (IS), defined as learners’ envisioned of becoming competent future language users, is one of the subdimensions of the Second Language Motivational Self System (L2MSS; Dörnyei, 2009). It serves as a core predictor of motivated behavior (Feng and Papi, 2020; Li and Zhang, 2021) and exerts explanatory power for L2 proficiency (Liu and Thompson, 2018). Learners with high IS show a higher tendency to strive to narrow the gap between their current selves and envisioned future selves, which is reflected in engaged behaviors and diligent efforts in daily tasks (Liu and Thompson, 2018). On the other hand, according to the domain-specific language mindset theory (Lou and Noels, 2017), language mindset, including growth mindset and fixed mindset, has also been claimed to be linked with learning engagement (Fan et al., 2024). Individuals who endorse a growth mindset are more likely to hold malleable beliefs and think that their language ability can be enhanced through practice and hard work. Therefore, they are willing to invest effort in engaging in learning-related activities (Lou and Noels, 2017).

The theoretical relationship among IS, mindset, and engagement is supported by the social cognitive theory (SCT; Bandura, 1986) and the Language-Mindset Meaning System (LMMS; Lou and Noels, 2019). Specifically, SCT posits that social factors, such as personal beliefs and thoughts, can influence one’s learning actions. Prior research has confirmed that language learners who have a clear image of their future competent selves (IS) tend to invest more effort in learning the target language (Feng and Papi, 2020; Li and Zhang, 2021). Students with an incremental mindset are more likely to engage more in learning tasks and use more strategies (e.g., planning and self-monitoring; Bai and Guo, 2021). On the other hand, the LMMS examines how language mindset contributes to language learning motivation. It suggests that language mindsets are foundational to the meaning-making processes that help learners make sense of their language learning experiences. Students who hold a growth mindset show heightened motivation and are more inclined to engage in learning (Lou and Noels, 2019).

Empirically, a plethora of previous studies have been dedicated to identifying the interwoven effects of IS, language mindset, and learning engagement in recent years (e.g., Sun et al., 2024; Xu et al., 2025). However, they either examined these constructs in isolation or adopted different configurations and methodological approaches. For example, Zhan and Zhong (2025) applied latent profile analysis (LPA), profiling IS and grit factors and examined their effects on engagement across different profile groups. Xu et al. (2025) used structural equation modeling and built a framework linking mindset, motivation, strategy use, and language performance. Ebn-Abbasi et al. (2024) examined the mediating effects of growth mindset and ideal L2 self on L2 grit and willingness to communicate. Yet, the integration of IS, mindset, and engagement within a single configuration, with mindset functioning as a mediator, still needs further exploration. Grounded in the theoretical frameworks of SCT and LMMS and previous empirical studies, the study intends to unveil the predictive effects of ideal L2 self on behavioral, emotional, and cognitive engagement, focusing on mindsets as mediators to capture the contributing factors influencing language engagement during the language learning process.

## 2 Literature review

### 2.1 Ideal L2 self and learning engagement

The L2MSS developed by Dörnyei (2005, 2009) is a widely used theoretical framework to gage language learners’ motivational levels. It was originally derived from two overarching theories: the possible selves theory by Markus and Nurius (1986) and the self-discrepancy theory by Higgins (1987). It contains three subscales: ideal L2 self (envisioning being a competent and successful learner), ought-to L2 self (reflecting expectations from significant others), and language learning experience (referring to immediate learning environments and past experiences). A volume of extant studies have posited that IS is a salient predictor of motivated learning behavior/intended learning efforts (Al-Hoorie, 2018; Feng and Papi, 2020; Li and Zhang, 2021) and has exerted significant effects on L2 achievement (Liu and Thompson, 2018).

Language learning engagement is a multifaceted construct, conceptualized as learners’ active involvement and participation in tasks (Hiver et al., 2024). Fredricks et al. (2004) delineate it into behavioral engagement (physical participation in academic and social activities), emotional engagement (affective reactions, both positive and negative, toward learning environments), and cognitive engagement (willingness and thoughtfulness in processing complex ideas and solving challenging tasks). Many studies have reported that engagement is a prerequisite for L2 achievement (Li and Yuan, 2024; Luan et al., 2025; Tsao et al., 2021).

Numerous prior studies have demonstrated that ideal L2 self is conducive to enhancing learners’ engagement (Abdollahzadeh et al., 2022; Tsao et al., 2021). For example, Zhan and Zhong (2025) profiled 479 university students majoring in languages. They reported that learners with a high ideal L2 self and low consistency of interest scored higher than those with a low ideal L2 self and low consistency of interest in emotional and behavioral engagement. Similarly, Tsao et al. (2021) verified the relationship between motivation, learner engagement, and writing proficiency. They discovered that ideal L2 self significantly promoted learners’ engagement and directly enhanced writing performance. The link was further highlighted by Abdollahzadeh et al. (2022), who examined the effects of motivation on engagement in academic reading among a group of undergraduate students. The findings showed that ideal L2 self was predictive of transformative engagement. Zhang et al. (2020) explored the connections between motivation, class engagement, L2 learning anxiety, and language achievement among 591 tertiary-level students; the results suggested that ideal L2 self directly and significantly predicted classroom engagement. In light of the above empirical studies, we proposed our first hypothesis (H1a-H1c) as follows:

*H*1: Ideal L2 self positively predicts learning engagement.

### 2.2 Language mindset and learning engagement

Mindset is defined as learners’ implicit beliefs about the nature of their intelligence. It entails both a growth and fixed mindset (Dweck, 2006). Learners endorsing a growth mindset view their abilities as malleable, while those endorsing a fixed mindset tend to think of their abilities as immutable and predetermined (Lou et al., 2021). Recent studies of mindset in SLA have attracted increasing scholarly attention (e.g., Jiang et al., 2024; Lou et al., 2021; Xu and Feng, 2024), and previous research has reported positive associations between language mindset and L2 learners’ motivated behaviors, such as willingness to communicate (Zhang et al., 2022), learning engagement (Guan et al., 2024), and self-regulated learning strategy use (Bai and Wang, 2023). Additionally, some empirical studies have demonstrated that language mindset, particularly a growth mindset, promotes L2 achievement (Derakhshan and Fathi, 2024b; Jiang et al., 2024).

Prior studies have attempted to prove the link between growth mindset and learning engagement. For example, Zhong et al. (2023) examined 1,738 Chinese undergraduates from two universities and tested the relationships among language mindset, positive and negative emotions, and engagement. The findings indicated that a growth mindset positively predicted learners’ engagement. A similar relationship was also presented in the study by Jiang et al. (2024), who recruited 486 high school learners and examined the structural interconnections among mindset, autonomous motivation, learning engagement, and language performance. The results suggested a positive link between growth mindset and engagement (*r* = 0.32). Similarly, Lou et al. (2021) profiled 234 undergraduate learners to examine distinct mindset groups and their associations with engagement and language achievement. The findings indicated that the high growth mindset profile group was the mostly engaged and also achieved the highest test scores.

However, recent research on structural relationships involving a fixed mindset remains sparse and limited, as most existing studies have focused solely on growth mindset (e.g., Bai and Wang, 2023; Derakhshan and Fathi, 2024b; Fan et al., 2024; Jiang et al., 2024; Sadoughi and Hejazi, 2023; Zhong et al., 2023). Since a fixed mindset is an integral component of mindset theory, it can guide how people think, feel, and act across different domains (Dweck, 2006). For example, in educational settings, learners with a fixed mindset are motivated to validate their competence because they believe their ability is innate, so they tend to avoid challenging situations (Lou and Noels, 2019). Additionally, according to the LMMS framework (Lou and Noels, 2019), growth mindset and fixed mindset are two separate meaning systems; each subsystem consists of a set of related cognitive and emotional factors that work together to create consistent differences in key motivational processes. Thus, the two scales cannot be examined separately. Recent empirical studies also report that fixed mindset exerts statistical influences on learners’ behaviors (Eren and Rakıcıoğlu-Söylemez, 2023; Guan et al., 2024). For example, Eren and Rakıcıoğlu-Söylemez (2023) investigated the associations of mindsets, engagement, instrumentality, and performance among 526 Turkish university EFL learners. The findings uncovered a negative association between fixed mindset and four dimensions of engagement. From both theoretical and practical perspectives, the scale of fixed mindset should be incorporated into the structural model. The second hypothesis (H2a-H2f) is proposed as follows:

*H*2: Growth mindset positively predicts engagement, and fixed mindset negatively predicts engagement.

### 2.3 Ideal L2 self and language mindset

The connection between ideal L2 self and mindset in L2 education research has been shown to be related (e.g., Yao et al., 2024; Xu and Wang, 2022). Learners with a growth mindset are more prone to picture themselves as proficient language users. The theoretical link was supported by the Language-Mindset Meaning System (LMMS) proposed by Lou and Noels (2019), which highlights the central role of language mindset and its influence on learners’ motivational beliefs. It claims that both growth and fixed mindsets are systematically linked to distinct motivational beliefs and guide learners to develop different affective and behavioral coping strategies.

Many extant studies have examined the associations between ideal L2 self and language mindset (e.g., Ebn-Abbasi et al., 2024; Yao et al., 2024). For example, Xu and Wang (2022) recruited 362 EFL undergraduates to investigate the nexus between growth mindsets, future writing selves, and self-regulated writing strategies. Their findings indicated that a growth mindset was a positive predictor of ideal L2 self (*r* = 0.31, *p* < 0.01). Similarly, Yao et al. (2024) examined 679 12th graders and found that growth mindset among both male and female groups positively predicted ideal writing self (*r* = 0.52 and *r* = 0.68, respectively). This connection was also observed in the study by Sadoughi et al. (2023). They explored the predictive effects of growth mindset on academic engagement, with future self-guides and language learning experience working as mediators. It was found that growth mindset positively and directly predicted ideal L2 self (*r* = 0.36, *p* < 0.01). With regard to the effects of fixed mindset on ideal L2 self, Kırmızı et al. (2023) reported that fixed mindset showed a negative association with ideal L2 self. As such, we proposed our third hypothesis (H3a-H3b) as follows:

*H*3: Ideal L2 self positively predicts growth mindset but negatively predicts fixed mindset.

### 2.4 The relationship among ideal L2 self, language mindset, and learning engagement

The relationship among ideal L2 self, language mindset, and engagement has been verified by a volume of prior research. However, existing studies tend to explore the constructs through different configurations and mediational pathways. For instance, numerous studies have conducted structural equation modeling to examine the influence of a growth mindset on engagement, with attribution (Guan et al., 2024), boredom (Derakhshan et al., 2022), and positive and negative emotions (Zhong et al., 2023) serving as mediators. In a similar vein, the effects of ideal L2 self on engagement also involve different mediating variables, such as L2 motivation (Tsao et al., 2021) and grit (Derakhshan and Fathi, 2024a). Additionally, several other researchers have explored the structural relationships between mindset, engagement, and L2 attainment (Eren and Rakıcıoğlu-Söylemez, 2023; Jiang et al., 2024; Lou et al., 2021; Xu and Feng, 2024). The findings from the above studies tended to report positive associations among the variables examined.

However, to date, empirical research has rarely examined ideal L2 self, language mindset, and engagement within a unified framework, particularly with both dimensions of mindset operating as mediators. To our knowledge, only one study by Sadoughi et al. (2023) addressed these three constructs from a holistic perspective. They specified the predictive pathway from growth mindset to engagement through ideal L2 self and identified statistically positive relationships among the involved variables. Considering that the three constructs represent pivotal components in SLA and have been shown to be interrelated and mutually affected, it is imperative to examine their collective effects and clarify the nature of their interactions. Accordingly, the present study advances the fourth hypothesis (H4a-H4f):

*H*4: Language mindset mediates the relationship between ideal L2 self and learning engagement.

### 2.5 The hypothesized model and research questions

**Figure 1:**
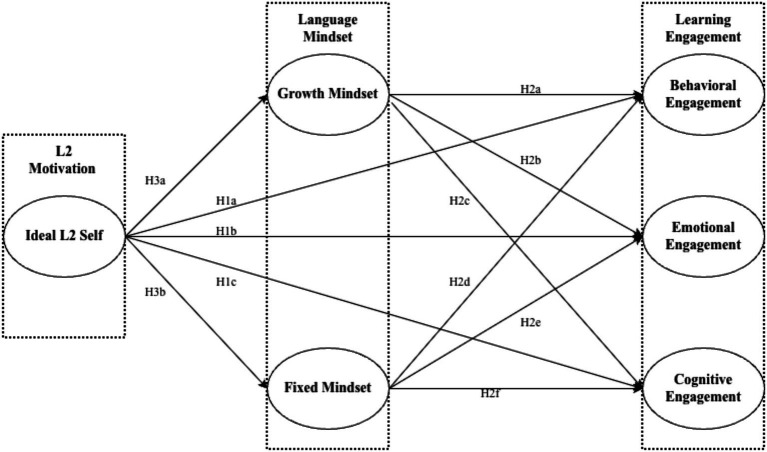
Research model.

To address the aforementioned gap, the current study structured ideal L2 self as an antecedent variable, two dimensions of language mindsets as mediators, and three dimensions of the language engagement scale as outcome variables (see Figure 1). We aim to address the following research questions:

RQ1: What is the structural relationship among ideal L2 self, language mindset, and learning engagement?

RQ2: How does language mindset mediate the relationship between ideal L2 self and learning engagement?

## 3 Method

### 3.1 Participants and data collection procedure

Adopting a convenience sampling method, 504 university students answered the survey. After deleting outliers and responses with incorrect student number formats, a final 431 valid responses were retained (male students = 62; female students = 369), with an average age of 21.3 years (M = 21.3, SD = 1.16). Considering the gender imbalance, we performed an independent sample *t*-test to examine whether there were any gender differences in the variables involved. The results showed that no differences were observed in ideal L2 self, fixed and growth mindsets, and behavioral engagement. Emotional and cognitive engagement showed minimal gender effects (see Table 1).

**Table 1:** Independent sample *t*-test.

Variables	Female		Male		t	*p*	Boot 95% CI
	Mean	SD	Mean	SD			
IS	3.331	0.681	3.31	0.666	0.223	0.823	[−0.154, 0.204]
FM	2.387	0.786	2.411	0.814	−0.230	0.822	[−0.241, 0.195]
GM	3.970	0.552	3.866	0.629	1.233	0.221	[−0.059, 0.272]
BE	3.713	0.533	3.625	0.521	1.203	0.230	[−0.053, 0.229]
EE	3.578	0.540	3.415	0.536	2.195	*	[0.014, 0.312]
CE	3.510	0.556	3.301	0.716	2.623	*	[0.026, 0.400]

*p < 0.05; IS, ideal L2 self; GM, growth mindset; FM, fixed mindset; BE, behavioral engagement; EE, emotional engagement; CE, cognitive engagement.

Learners take 2 years of comprehensive college English courses, with an approximately 16-week teaching schedule each semester. There are four lessons per week, and each lesson lasts 45 min. Ethics approval was first secured from the first author’s university, after which the corresponding author, who is also a lecturer at the target university, assisted with data collection. The questionnaire link and QR code generated via the *Wenjuanxing* platform were first disseminated to students taught by the corresponding author and then shared with other lecturers, who distributed the questionnaire to their respective classes. Participants were notified in advance that their involvement was voluntary and that all data gathered would be utilized exclusively for research purposes.

### 3.2 Instruments

The survey instrument consists of two parts. The first part gathered participants’ background information, including age, gender, and student identification numbers. The second part measured learners’ motivational orientations, language mindset, and levels of learning engagement. The questionnaire used a 5-point Likert scale (1 = *strongly disagree* to 5 = *strongly agree*) and underwent a translation and back-translation procedure to ensure that the original English version was accurately rendered for the participants. In addition, two qualified teachers independently reviewed the translated questionnaire, provided feedback, and resolved any discrepancies. Minor revisions were subsequently made.

Learning engagement. The learning engagement construct was adapted from Li et al. (2024) to gage students’ levels of engagement. The original engagement construct by Li et al. (2024) encompasses four dimensions. However, prior research has suggested that social engagement overlaps with behavioral engagement (Oga-Baldwin, 2019). Accordingly, we adopted only three dimensions. The current engagement construct contains 14 items across three subscales. After eliminating items with factor loadings less than 0.6, we ultimately retained 11 items. Specifically, behavioral engagement included four items (e.g., “If there is anything that I do not understand in English class, I will figure it out after class”), emotional engagement consisted of four items (e.g., “When we do activities in English class, I feel interested”), and cognitive engagement comprised three items (e.g., “My mind seldom wanders in English class”). Cronbach’s alpha results confirmed that each subscale achieved acceptable levels of internal consistency (0.823, 0.829, and 0.840, respectively).

Ideal L2 self. The ideal L2 self scale was derived from Dörnyei (2009) to measure students’ motivation levels related to their future competent and successful selves. The scale contains five items, with one item removed due to low factor loading. The Cronbach’s alpha for the four retained items (e.g., “I can imagine myself living abroad and using English effectively”) is 0.827.

Language mindset. The language mindset construct was adapted from Lou and Noels (2017) to measure learners’ mindset levels. The construct comprises 10 items with 5 items for the growth mindset and 5 items for fixed mindset. After deleted items that obscured the factor structure, as well as those with factor loadings below 0.6. We finally retained three items tapping growth mindset (e.g., “I can improve my English ability through effort and practice”) and four items rating fixed mindset (e.g., “I have a certain amount of English ability, and I can’t really do much to change it”). The Cronbach’s alpha coefficients for growth and fixed mindset subscales are 0.782 and 0.873, respectively.

### 3.3 Data analysis

The data were analyzed using SPSS 27 and AMOS 27 in three sequential stages. Initially, model fit indices were examined to evaluate the adequacy of the measurement model and the validity of the constructs. Then, descriptive statistics were calculated to summarize the dataset, followed by correlation analyses to explore associations among variables. Structural equation modeling (SEM) was lastly applied to test the proposed hypotheses. To further assess the significance of indirect effects, bootstrapping procedures with user-defined estimations in AMOS 27 were employed to verify the mediation pathways.

## 4 Results

### 4.1 Confirmatory factor analysis

Table 2 presents the evidence of construct validity for IS, language mindset, learning engagement, and the overall model. Construct validity was examined from three perspectives: model fit, convergent validity, and discriminant validity. As indicated in Table 2, the structural model fit indices for all measurement models met the recommended benchmarks proposed by Marsh et al. (2004) (see Table 2). In terms of convergent validity, both CR and AVE for IS, mindset, and engagement were above the recommended cutoff values of 0.70 and 0.50, respectively (Marsh et al., 2004).

**Table 2:** Goodness-of-fit indices and convergent validity.

Variables	Goodness-of-fit indices					Convergent validity	
	X^2^/df	CFI	TLI	RMSEA	SRMR	CR	AVE
Benchmark	<5	>0.900	>0.900	<0.008	<0.100	>0.700	>0.500
Ideal L2 self	2.308	0.998	0.988	0.055	0.008	0.832	0.555
Mindset	1.273	0.997	0.995	0.025	0.014	-	-
Growth mindset	-	-	-	-	-	0.767	0.523
Fixed mindset	-	-	-	-	-	0.874	0.634
Engagement	2.828	0.967	0.956	0.065	0.017	-	-
Behavioral engagement	-	-	-	-	-	0.824	0.539
Emotional engagement	-	-	-	-	-	0.842	0.571
Cognitive engagement	-	-	-	-	-	0.844	0.643
Overall model	2.998	0.915	0.900	0.063	0.068	-	-

df, degree of freedom; CFI, comparative fit index; TLI, Tucker–Lewis index; RMSEA, root mean square error of approximation; SRMR, standardized root mean square residual; CR, composite reliability; AVE, average variance extracted.

Table 3 reports the results of the discriminant validity analysis for the measured variables. It is clear that the square root of the AVE for each construct exceeded the corresponding inter-construct correlations, which satisfies the benchmark proposed by Hiver and Al-Hoorie (2020), meaning that the measured constructs are conceptually distinct.

**Table 3:** Discriminant validity.

Variables	1	2	3	4	5	6
1. Ideal L2 self	**0.745**					
2. Fixed mindset	−0.117	**0.796**				
3. Growth mindset	0.281	−0.033	**0.723**			
4. Cognitive engagement	0.515	−0.026	0.496	**0.802**		
5. Emotional engagement	0.698	−0.110	0.553	0.495	**0.756**	
6. Behavioral engagement	0.581	−0.084	0.663	0.490	0.600	**0.734**

The bold represents the square root of AVE.

### 4.2 Descriptive statistics and correlation analysis

Before moving to the main analysis, Harman’s single-factor test was conducted to assess common method bias (Podsakoff et al., 2003). The results revealed that five factors had eigenvalues greater than 1, of which the first factor contributed to 33.98% of the total variance, below the threshold of 40%. This suggests that common method bias is not a serious concern in the present study.

Table 4 provides the descriptive statistics and correlation matrix for the involved variables. Among the constructs, growth mindset recorded the highest mean score (M = 3.955, SD = 0.565), while the mean score for fixed mindset saw the lowest (M = 2.390, SD = 0.789). Normality was assessed following Collier (2020) guideline (−2 < skewness and kurtosis < 2). As presented in Table 4, the skewness and kurtosis values were all within the acceptable range. As for the correlation within variables, the correlation coefficients showed that apart from fixed mindset, which did not show a statistically significant relationship with ideal L2 self, all the other variables were interrelated with moderate strength.

**Table 4:** Descriptive statistics and correlation analysis.

Variables	1	2	3	4	5	6	Mean	SD	Skewness	Kurtosis
1. IS	-						3.328	0.678	−0.578	0.278
2. GM	0.245**	-					3.955	0.565	−0.307	0.894
3. FM	−0.071	−0.317**	-				2.390	0.789	0.544	−0.179
4. BE	0.457**	0.488**	−0.193**	-			3.700	0.532	−0.090	0.197
5. EE	0.568**	0.404**	−0.182**	0.600**	-		3.555	0.542	−0.196	0.169
6. CE	0.394**	0.308**	−0.097*	0.530**	0.642**	-	3.480	0.585	−0.556	0.848

*p < 0.05; **p < 0.01.

### 4.3 The structural model

**Figure 2:**
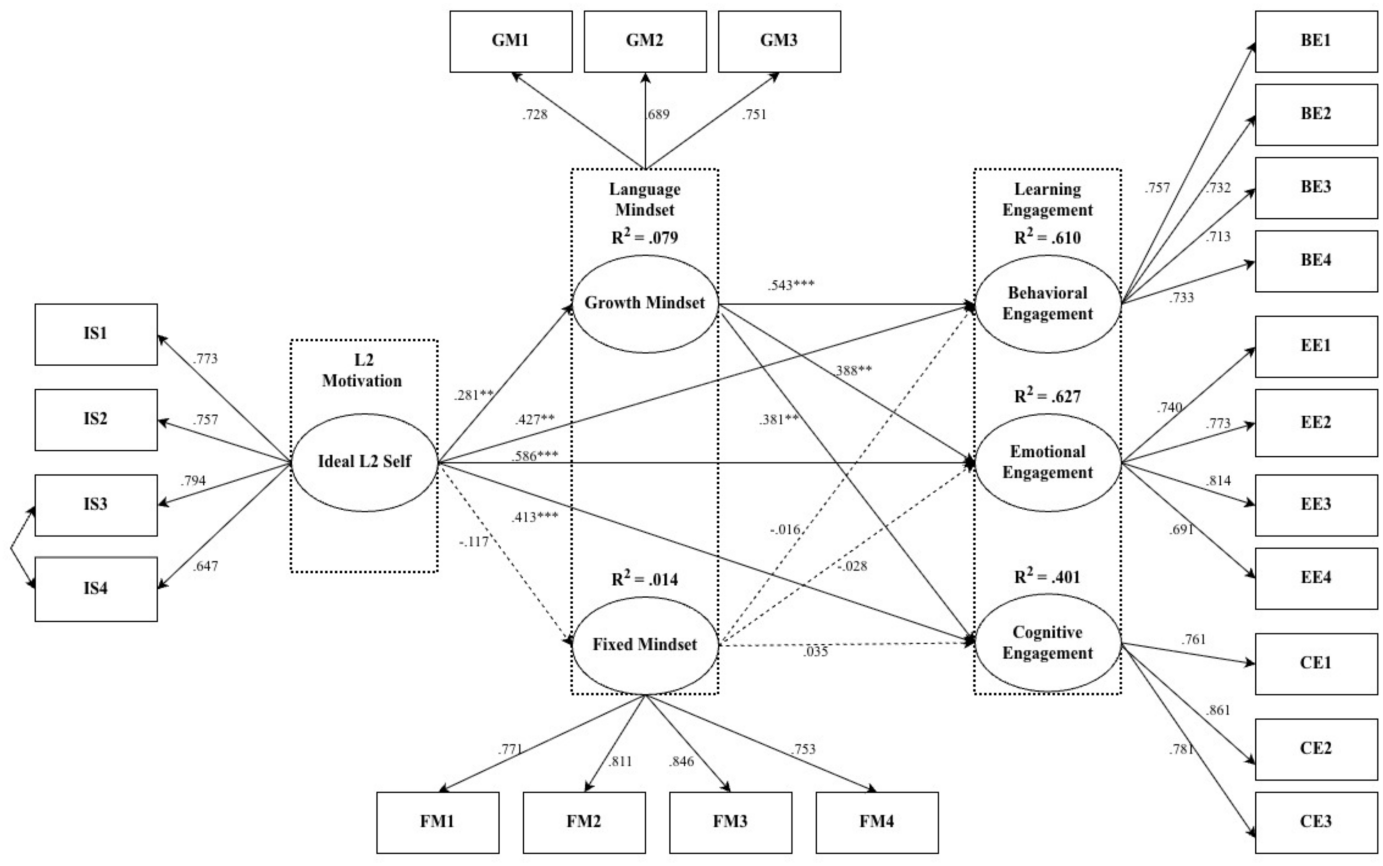
Verification of the structured model.

SEM was computed to examine the hypothesized connections. After deleting outliers and factor loadings below 0.6 (see Figure 2), 22 items remained. The overall fit indices were χ^2^/df = 2.998, CFI = 0.915, TLI = 0.900, RMSEA = 0.063, and SRMR = 0.068, which satisfied the criteria proposed by Marsh et al. (2004).

#### 4.3.1 Direct effect analysis

As shown in Table 5, the direct effects were measured through Amos 27. Regarding H1, ideal L2 self significantly and positively predicted behavioral, emotional, and cognitive engagement, with standardized coefficients of *β* = 0.427 (*p* < 0.01), *β* = 0.586 (*p* < 0.001), and *β* = 0.413 (*p* < 0.001), respectively. As for H2, the growth mindset also showed significant positive effects on the three dimensions of engagement. Specifically, it statistically predicted behavioral (*β* = 0.543, *p* < 0.001), emotional (*β* = 0.388, *p* < 0.01), and cognitive engagement (*β* = 0.381, *p* < 0.01). In contrast, fixed mindset did not serve as a statistical predictor for the three facets of engagement. Concerning the effects of ideal L2 self on mindset (H3), the direct path analysis results demonstrated that IS positively predicted growth mindset (*β* = 0.281, *p* < 0.01) but did not function as a significant facilitator of fixed mindset. Overall, IS explains 9.3% of the variance for language mindset (7.9% for growth mindset and 1.4% for fixed mindset). Ideal L2 self and language mindset accounted for 61.0% of the variance in behavioral engagement, 62.7% in emotional engagement, and 40.1% in cognitive engagement (see Figure 2).

**Table 5:** Direct and indirect effects.

Hypotheses		Pathway	β	SE	95% CI (bias-corrected)		*p*
					Lower	Upper	
Direct effects							
H1	H1a	IS→BE	0.427	0.064	0.291	0.544	**
	H1b	IS→EE	0.586	0.065	0.448	0.701	***
	H1c	IS→CE	0.413	0.082	0.246	0.562	***
H2	H2a	GM → BE	0.543	0.069	0.411	0.688	***
	H2b	GM → EE	0.388	0.081	0.244	0.569	**
	H2c	GM → CE	0.381	0.092	0.216	0.585	**
	H2d	FM → BE	−0.016	0.046	−0.104	0.078	0.739
	H2e	FM → EE	−0.028	0.053	−0.132	0.075	0.578
	H2f	FM → CE	0.035	0.063	−0.092	0.153	0.592
H3	H3a	IS→GM	0.281	0.053	0.174	0.382	**
	H3b	IS→FM	−0.117	0.062	−0.235	0.006	0.060
Indirect effects							
H4	H4a	IS→GM → BE	0.152	0.036	0.095	0.238	***
	H4b	IS→GM → EE	0.109	0.032	0.064	0.195	***
	H4c	IS→GM → CE	0.107	0.035	0.060	0.203	***
	H4d	IS→FM → BE	0.002	0.006	−0.009	0.017	0.562
	H4e	IS→FM → EE	0.003	0.007	−0.008	0.023	0.415
	H4f	IS→FM → CE	−0.004	0.009	−0.029	0.009	0.419

**p < 0.01, ***p < 0.001. IS, ideal L2 self; GM, growth mindset; FM, fixed mindset; BE, behavioral engagement; EE, emotional engagement; CE, cognitive engagement.

#### 4.3.2 Indirect effect analysis

Concerning the indirect effects of ideal L2 self on engagement (H4), the self-defined estimation through Amos with bootstrapping 5,000 samples revealed that growth mindset greatly mediated the relationship between ideal L2 self and the three dimensions of learning engagement. The mediating effects were *β* = 0.152 and *p* < 0.001 for behavioral engagement, *β* = 0.109 and *p* < 0.001 for emotional engagement, and *β* = 0.107 and *p* < 0.001 for cognitive engagement, accordingly. In contrast, fixed mindset did not show any direct or indirect statistical significance, neither with ideal L2 self nor with learning engagement (see Table 5).

## 5 Discussion

### 5.1 The effects of ideal L2 self on learning engagement

The results showed that ideal L2 self serves as a direct facilitator of students’ learning engagement (H1), meaning that learners with a better image of their competent future selves would display active involvement in tasks, affective reactions toward learning environments, and willingness to face challenging activities. The finding echoes some prior studies asserting that students’ positive image of their better future selves as catalysts for facilitating engagement (Abdollahzadeh et al., 2022; Tsao et al., 2021; Zhang et al., 2020). The results were also consistent with numerous studies claiming that ideal L2 self is a salient predictor of learners’ intended learning effort/motivation intensity (similar to the scale of behavioral engagement; e.g., Al-Hoorie, 2018; Li and Zhang, 2021). Additionally, the study further confirmed the positive psychology vogue, reporting that learners who have clear goals in mind tend to be more capable of adjusting to challenging situations and thus actively invest more effort to achieve their imagined L2 learning goals (Zarrinabadi et al., 2022). Thus, to better promote students’ learning engagement, more attention should be given to enhancing learners’ ideal L2 self.

### 5.2 The effects of language mindset on learning engagement

Learners’ growth mindset positively predicts the three dimensions of learning engagement, indicating that students who believe their language intelligence is malleable and can be enhanced through sustained endeavors are more inclined to be behaviorally, emotionally, and cognitively engaged in learning tasks. The results resonate with previous studies by Zhong et al. (2023), Jiang et al. (2024), and Lou et al. (2021). The findings also align with social cognitive theory, which posits that personal beliefs are critical determinants of learning behavior (Bandura, 2001) and further corroborate the salient predictive role of growth mindset in learners’ motivation levels (Lou and Noels, 2016).

On the other hand, fixed mindset did not demonstrate statistical significance in predicting learning engagement, suggesting that students who believe their language intelligence is innate or predetermined did not exhibit any association with their actual behaviors. This finding contradicts some prior studies reporting that fixed mindset negatively or indirectly influences learning engagement (Eren and Rakıcıoğlu-Söylemez, 2023; Guan et al., 2024). A possible explanation may be attributed to the multifaceted nature of language learning engagement (Hiver et al., 2024), which can be affected by a constellation of factors, such as resilience (Xu and Feng, 2024) and emotions (Liang, 2025), instead of a single factor. Additionally, the insignificant relationship between fixed mindset and engagement echoes the claim by Xu and Feng (2026), which reports that fixed mindset alone does not inflict statistical effects on engagement (see the results of the tested model one for review), but along with growth mindset that together affect engagement in non-linear ways. The results highlight that fixed and growth mindsets should not be examined separately.

### 5.3 The effects of ideal L2 self on language mindset

Learners’ ideal L2 self is a positive predictor of growth mindset. The finding echoes a volume of extant studies (Sadoughi et al., 2023; Xu and Wang, 2022; Yao et al., 2024) and further provides evidence bridging the gap between the two popular motivational frameworks. The results also resonate with the LMMS framework, suggesting that language mindsets are intertwined with learners’ motivation and impact how learners feel, think, and act toward target goals (Lou and Noels, 2019). However, ideal L2 self did not statistically relate to fixed mindset. The result contradicts one previous study positing a negative relationship between the two variables (Kırmızı et al., 2023). This is understandable due to the fact that some learners can have a vivid image of being proficient language users while still believing their language ability is rather fixed. Since only a sparse number of recent studies have involved fixed mindset and its relationship with other variables, more empirical research is warranted.

### 5.4 The mediating effects of mindset on ideal L2 self and engagement

Learners’ growth mindset was reported to positively mediate the relationship between ideal L2 self and learning engagement, while fixed mindset did not reach statistical significance. This finding indicates that learners with a positive version of their better future L2 selves are more inclined to develop incremental beliefs about their language ability, which in turn facilitates behavioral, emotional, and cognitive engagement in learning tasks. In contrast, learners who take entity beliefs did not appear to function as an explanatory mechanism connecting ideal L2 self to engagement. To date, a limited number of studies have examined the mediating role of language mindset in the link between ideal L2 self and learning engagement, and empirical evidence regarding the mediating role of fixed mindset remains especially scarce in the SLA domain. Since fixed and growth mindsets are not two sides of the same coin (Lou et al., 2021), previous studies have also confirmed that the two mindsets combined can jointly influence engagement (Xu and Feng, 2024, 2026). Thus, future studies regarding the mediating role of both mindset dimensions are encouraged.

## 6 Conclusion

The study unveiled the structural relationships among ideal L2 self, language mindset, and learning engagement among tertiary-level university students. The results show that ideal L2 self serves as both a direct and an indirect predictor of all three subcomponents of learning engagement. In addition, growth mindset is a significant mediator in the relationship between ideal L2 self and learning engagement, whereas fixed mindset did not present any statistically significant mediating effect between the two variables.

The study increased our understanding of positive beliefs and their predictive effects on engagement, providing further evidence for the positive psychology movement exploring positive psychological traits and their influence on achievement in recent studies. Based on the empirical study findings, we call for more attention to be given to language learners to foster the belief that their language ability can be enhanced through accumulated effort and sustained endeavor, that the improvement of their ability is not impeded by age or innate intelligence, and that positive beliefs are of great significance in fostering their learning engagement to optimize the learning process. Additionally, the study offers pedagogical guidance for teachers to plan and deliver their lessons, such as setting visualized goals, cultivating positive beliefs, and encouraging learners to adopt a growth mindset, which have been proven to be helpful in facilitating learning engagement and further enhancing language achievement in the long term.

However, the study has several limitations. First, the cross-sectional design has limitations in drawing accurate conclusions about motivational dynamics since motivation and language mindset are fluid processes that may change over time (Dörnyei, 2014). Future longitudinal research and intervention studies that aim to capture these changing patterns are encouraged. Second, even though common method bias was statistically checked and found not to be a serious issue, the reliance on a self-reported questionnaire collected from a single university may still introduce potential bias. A longitudinal or mixed-methods design with multiple sample sites is needed. Third, considering that the context is limited to China with only one university, learners from varying regional and cultural contexts may present distinct patterns. Since learning engagement is highly context-based in nature (Hiver et al., 2024), future studies involving diverse samples from different regions and age groups (e.g., learners from primary or secondary schools) are encouraged.

## Data Availability

The raw data supporting the conclusions of this article will be made available by the authors, without undue reservation.
